# A systematic review of fear of falling and related constructs after hip fracture: prevalence, measurement, associations with physical function, and interventions

**DOI:** 10.1186/s12877-023-03855-9

**Published:** 2023-06-23

**Authors:** Chandini Gadhvi, Debbie Bean, David Rice

**Affiliations:** 1 Health & Rehabilitation Research Institute, Auckland University of Technology, Auckland, New Zealand; 2 Allied Health - Physiotherapy, Te Whatu Ora Health New Zealand - Te Toka Tumai, Auckland, New Zealand; 3 Department of Anaesthesiology & Perioperative Medicine, Te Whatu Ora Health New Zealand - Waitematā, Auckland, New Zealand

**Keywords:** Fear of falling, Falls efficacy, Balance confidence, Hip fracture, Neck of femur fracture OR nof, Rehabilitation, Older adults

## Abstract

**Background:**

Hip fracture is a common and debilitating injury amongst older adults. Fear of falling (FoF) and related constructs (balance confidence and falls efficacy) may impede rehabilitation after hip fracture. An updated systematic review to synthesize existing literature on FoF after hip fracture is needed. This review focussed on four research questions: In the hip fracture population: (1) What is the prevalence of FoF?; (2) What FoF assessment tools are validated? (3) What is the relationship between FoF and physical function?; (4) What interventions are effective for reducing FoF?

**Methods:**

A systematic search was undertaken in EBSCO Health, Scopus and PsychINFO in January 2021 (and updated December 2022) for articles on FoF after hip fracture. Data in relation to each research question was extracted and analysed. The quality of the studies was appraised using the ‘Risk of Bias Tool for Prevalence Studies’, ‘COSMIN Risk of Bias checklist for Patient-reported outcome measures’, modified version of the ‘Appraisal Tool for Cross-sectional studies’, and the ‘Cochrane Risk of Bias 2’ tools for each research question, respectively.

**Results:**

36 studies (37 articles) with 5099 participants were included (mean age 80.2 years and average 78% female). Prevalence rates for FoF after hip fracture ranged between 22.5% and 100%, and prevalence tended to decrease as time progressed post hip fracture. The ‘Falls Efficacy Scale – International’ (FES-I) and ‘Fear of Falling Questionnaire – Revised’ (FFQ-R) were found to be reliable, internally consistent, and valid tools in hip fracture patients. FoF after hip fracture was consistently associated with measures of physical function including balance, gait speed, composite physical performance measures and self-reported function. Ten of 14 intervention studies were considered high risk of bias. Exercise-based interventions with or without a psychological component were not effective in reducing FoF after hip fracture compared to a control condition.

**Conclusion:**

FoF is prevalent after hip fracture and is consistently associated with poorer physical function. Only two instruments (FES-I and FFQ-R) have been validated for measuring FoF in the hip fracture population. However, there remains a need for larger, higher quality randomised controlled trials targeting FoF after hip fracture in order to guide clinical practice.

**Trial registration:**

PROSPERO registration: CRD42020221836.

**Supplementary Information:**

The online version contains supplementary material available at 10.1186/s12877-023-03855-9.

## Background

Sustaining a hip fracture is a serious consequence of falls [1] and a leading cause of disability among older adults [2]. The impact of hip fracture is huge, with significant costs of treatment, rehabilitation, assistance and, in some cases, long-term care [1, 3]. Globally, each hip fracture can cost an estimated $10,075 USD for hospitalisation, and $43,669 for health and social care costs at 1 year [4]. Moreover, mental health and quality of life are severely impacted by hip fracture [2]. Rehabilitation after hip fracture is highly challenging, so identifying factors that may impede or facilitate rehabilitation would be of value.

Fear of falling (FoF) refers to “a lasting concern about falling that leads to an individual avoiding activities that he/ she remains capable of performing” [5, p.36[. It is often a consequence of a fall, and has been recognised as a factor that may limit function since the 1980s [6, 7]. FoF is often operationalised by two related constructs, ‘falls efficacy’ and ‘balance confidence’ [8–10]. The terms of FoF, falls efficacy and balance confidence have been used interchangeably in the literature [10, 11] and in line with previous work [12], the term FoF will be used as an umbrella term to encompass the three related constructs for the purposes of this review.

Although FoF is common amongst older adults, particularly after a fall, its prevalence is higher in those with a fall-related fracture [13], such as a hip fracture, likely because the individual has experienced such a severe consequence of falling. Bower et al. (2016) found elevated FoF affected 60.5% and 47% of participants at four and twelve weeks post hip fracture, respectively [14]. Also, FoF may be different after hip fracture because the patient has suddenly become restricted in their activities [15]. Bower et al. (2016) suggest that FoF may be transient or dynamic after hip fracture and may change as time lapses post-fracture [14]. However, no recent systematic reviews have evaluated the prevalence of FoF after hip fracture. FoF after hip fracture is clinically important, because it may influence functional recovery after hip fracture [16, 17]. FoF has been shown to be associated with functional performance and functional recovery [14, 18] and therefore may be a modifiable risk factor and target for intervention [14, 19].

There are several instruments available to measure FoF and related constructs, such as the ‘Fear of falling questionnaire – revised’ (FFQ-R) and the ‘Falls efficacy scale – international’ (FES-I), but most were developed and tested in the general older adult or falls population rather than in hip fracture patients. Recently, some studies have investigated the psychometric properties of FoF instruments in hip fracture patients specifically [20, 21]. It is important to assess this data to determine if instruments are appropriate for hip fracture patients, because FoF could manifest differently after hip fracture compared to FoF in those without a fracture.

Several studies have linked FoF to poorer physical or functional performance in hip fracture patients [22, 23]. For example, high FoF has been shown to predict poorer functional recovery [14], and poorer gait speed and balance [18]. As such, FoF may influence functional recovery after hip fracture and is a potentially modifiable factor worth addressing to improve outcomes [14, 19]. However, there have been no recent systematic reviews that collate these findings to inform clinical practice.

Given the growing understanding of FoF as a multi-factorial issue, both physical and psychological interventions may be needed [24–26]. In hip fracture patients, clinical trials have investigated a range of interventions for FoF, including exercise based and cognitive behavioural interventions [27–29]. Although a number of trials have been published recently, their findings appear disparate and clear clinical recommendations are lacking. Previous reviews [30–32] have evaluated interventions during hip fracture rehabilitation but none have focussed on FoF specifically. Therefore, there is a need to synthesize the findings of FoF intervention trials in hip fracture patients.

In summary, FoF appears to influence hip fracture rehabilitation, and addressing it may improve outcomes [16]. Consolidating our knowledge of the prevalence and measurement of FoF after hip fracture, how it influences physical performance as well as how best to address FoF in hip fracture rehabilitation is therefore necessary. Since the last systematic review on FoF in hip fracture [15], many new studies focusing on FoF after hip fracture have been published. Therefore, this systematic review will review current literature on four research questions:

1. What is the prevalence of FoF in patients after hip fracture?
2. What are the psychometric properties of the instruments used to measure FoF in the hip fracture patient population?
3. What is the association between FoF and measures of physical function or performance after hip fracture?
4. Which interventions are effective in reducing FoF after hip fracture?

## Methods

This systematic review was conducted in accordance with the Preferred Reporting Items for Systematic Reviews and Meta-analyses (PRISMA) guidelines [33]. A protocol for this review was developed and registered on PROSPERO (CRD:42020221836).

### Search strategy

A systematic search was performed (by authors CG and DB) in January 2021 (with an updated search performed in December 2022) in the electronic databases of EBSCO Health Databases (including CINAHL Complete, MEDLINE and SPORTDiscus), Scopus, and PsychINFO for studies on FoF after hip fracture. The search terms and strategy were designed with input from a trained librarian. A detailed search strategy for each database is outlined in supplementary file 1.

### Inclusion and exclusion criteria

Identified studies were included if they: (1) included participants with a diagnosis of hip fracture, (2) measured FoF (including the related constructs falls efficacy or balance confidence), (3) had full-text available in English, and (4) answered one of the four research questions. Studies were excluded if they were: (1) not peer-reviewed, (2) not original research, (3) performed in a mixed population where independent data on hip fracture participants could not be extracted or obtained, (4) qualitative studies, (5) uncontrolled trials, (6) pilot or feasibility studies, and (7) studies that did not report their FoF data. The exclusion of pilot and feasibility studies (for research question 4) was added to the criteria after submission of the protocol on Prospero, but was deemed appropriate for this systematic review which focussed on treatment efficacy rather than feasibility.

### Study selection

The search strategy was applied to all databases by two authors (CG, DB) simultaneously. All identified studies were downloaded and duplicates were removed manually. The titles and abstracts were screened by two reviewers (CG and DB) independently according to the inclusion and exclusion criteria. The full-texts of all potentially eligible studies were screened. Disagreements on article inclusion/ exclusion were discussed and a third person (DR) was involved if an agreement could not be reached. The reference lists and forward citations (using Google Scholar and Scopus) of all included studies were searched to look for further relevant studies.

### Data extraction

Two reviewers independently extracted data from all included studies into a Microsoft Excel spreadsheet (CG and DB, questions 2 and 3; CG and DR, questions 1 and 4). The two reviewers discussed any disagreements and a third person (DB or DR) was involved if required. For each included study the following data were extracted: study design and details, sample size, participant characteristics (age, gender), days since hip fracture and FoF measure(s) used. Additionally, for research question 1, FoF prevalence; for research question 2, statistical data pertaining to internal consistency, reliability, validity and other related psychometric properties of outcome measures; for research question 3, outcome measure used for the comparator variable (physical functional or performance factors) and correlation or regression statistics measuring the association between the comparator variable and FoF; and for research question 4, intervention used and resulting FoF data comparing the intervention group with control group as well as drop-out rate, were extracted where applicable. For the purpose of question 3, physical function refers to the ability to perform basic actions essential for maintaining independence as well as carrying out more complex activities [34] and we included studies with any objective measure of physical function or patient self-reported measure of function. For question 4, any intervention modality was accepted as long as the study was a clinical trial and a measure of FoF was included. One randomised controlled trial (RCT) included mostly hip and some pelvic fracture patients [28]; this author was contacted and data specific to only the hip fracture participants included in their study were obtained.

### Quality and risk of bias appraisal

Each included study was appraised by two reviewers; any disagreements were resolved by involving the third reviewer. The four research questions were answered by studies of different designs; therefore, four quality assessment tools were required to appraise the included studies (one tool for each research question). Prevalence studies included to answer the first research question were appraised using the Risk of Bias Tool for Prevalence Studies [35] which is a 10 item tool assessing external and internal validity of the study across four domains of bias. Studies investigating psychometric properties of outcome measures were appraised using the COSMIN Risk of Bias checklist for Patient-reported outcome measures instruments [36]. A modified version of the Appraisal Tool for Cross-sectional studies (AXIS) tool [37] was used to appraise the cross-sectional and prospective longitudinal studies that were included to answer the third research question. The modification was that 3 items from the NIH Assessment Tool for Observational Cohort and Cross-Sectional Studies [38] were added to assess blinding, loss to follow-up and adjustment for confounders. Finally, clinical trials answering research question 4 were evaluated using the Cochrane Risk of Bias 2 tool, known as RoB2 [39].

### Data analysis

The data were analysed and synthesized for each of the four research questions separately, using a Microsoft Excel spreadsheet, and Microsoft Excel was also used for any effect size calculations. For research question 1, the extracted prevalence rates were analysed in relation to the time point at which they were measured. The data was graphed on a scatter plot with prevalence rate plotted against the time (in weeks) at which it was measured post hip fracture. When the prevalence rate was given for a time period, the mid-point of that time period was used to plot the prevalence rate. The range of prevalence rates for the following time periods post-fracture are also described in the text: 1–4 weeks, ~ 12 weeks and 12–58 weeks.

For research question 2, the data for each instrument were individually extracted and tabulated. The statistical values for each psychometric property were interpreted as follows. For test-retest reliability, the extracted intraclass correlation coefficient values were analysed as poor, moderate, good or excellent as outlined by Koo and Li [40]. Cronbach’s alpha coefficients for internal consistency were classed between ‘unacceptable’ to ‘excellent’ as outlined by George and Mallery [41]. Construct validity was described based on confirmation of ‘a priori’ hypotheses and strength of correlations with related constructs. Results from factor analysis were used to describe structural validity. Measurement error was interpreted as reported in the individual study.

Data extracted for studies in relation to question 3 were categorised based on the physical function or performance measure that FoF was associated with, which were: balance, gait speed, composite physical performance measure (i.e. measuring more than one aspect of physical performance), self-reported function, physical activity (e.g. step count), and muscle strength. For each category, the extracted statistical data was tabulated. Most studies reported Pearson or Spearman’s correlation coefficients measuring the association between FoF and physical function. The strength for each correlation coefficient was determined using Cohen’s guide: 0.10–0.29 is small, 0.30–0.49 is medium and ≥ 0.50 is large [42]. Some studies performed logistic regression analyses revealing an odds ratio (OR) for a dichotomous outcome; these were converted into an effect size (Cohen’s D or standardised mean difference) using the formula: ‘ln (OR) / 1.81’ [43]. Where the OR was less than 1, it was first converted into a number greater than 1 by using 1/OR to result in a positive number. The resulting effect size was interpreted using Cohen’s guide wherein 0.20 to 0.49 is considered a small effect size, 0.50 to 0.79 is medium and 0.80 and above is a large effect size [42]. A value below 0.20 was considered negligible. Some studies reported unstandardized or standardized beta coefficients from regression analyses. These were interpreted by taking the r^2^ to determine how much variance in the comparator variable was explained by the FoF variable [44] or by imputation of an r value from the standardised beta coefficient [45]. Only one study [23] performed a negative binomial or Poisson regression and reported an incidence rate ratio which was analysed as reported by the study. Finally, for each of the categories of physical function, the strength of the associations with FoF were summarised.

In order to analyse the effectiveness of interventions (for research question 4), between group effect sizes were calculated, where possible. Where means and standard deviations (SD) for the intervention group and control group were provided, a Cohen’s D effect size was calculated using the formula: ‘difference in means (intervention – control) / pooled SD’ [46]. Two studies [47, 48] provided median and range as raw data; this was converted to mean using the formula: ‘(minimum value + 2 x median + maximum value)/ 4’ and SD using the formula: ‘(maximum value – minimum value)/ 4’ as suggested by Hozo et al. [49], which were then converted into an effect size [46]. The effect size (standardised mean difference) was interpreted using Cohen’s guide as mentioned above [42]. One study provided only the between group differences [50] and one study [51] provided only median and 25th / 75th percentiles as raw data. An effect size could not be calculated for these studies; so only the statistical significance of their result was reported. Meta-analysis was not considered appropriate due to high risk of bias in a number of the included studies as well as substantial heterogeneity in the content and timing of the intervention, control group comparators, and time to follow up.

## Results

### Study selection

**Fig. 1:**
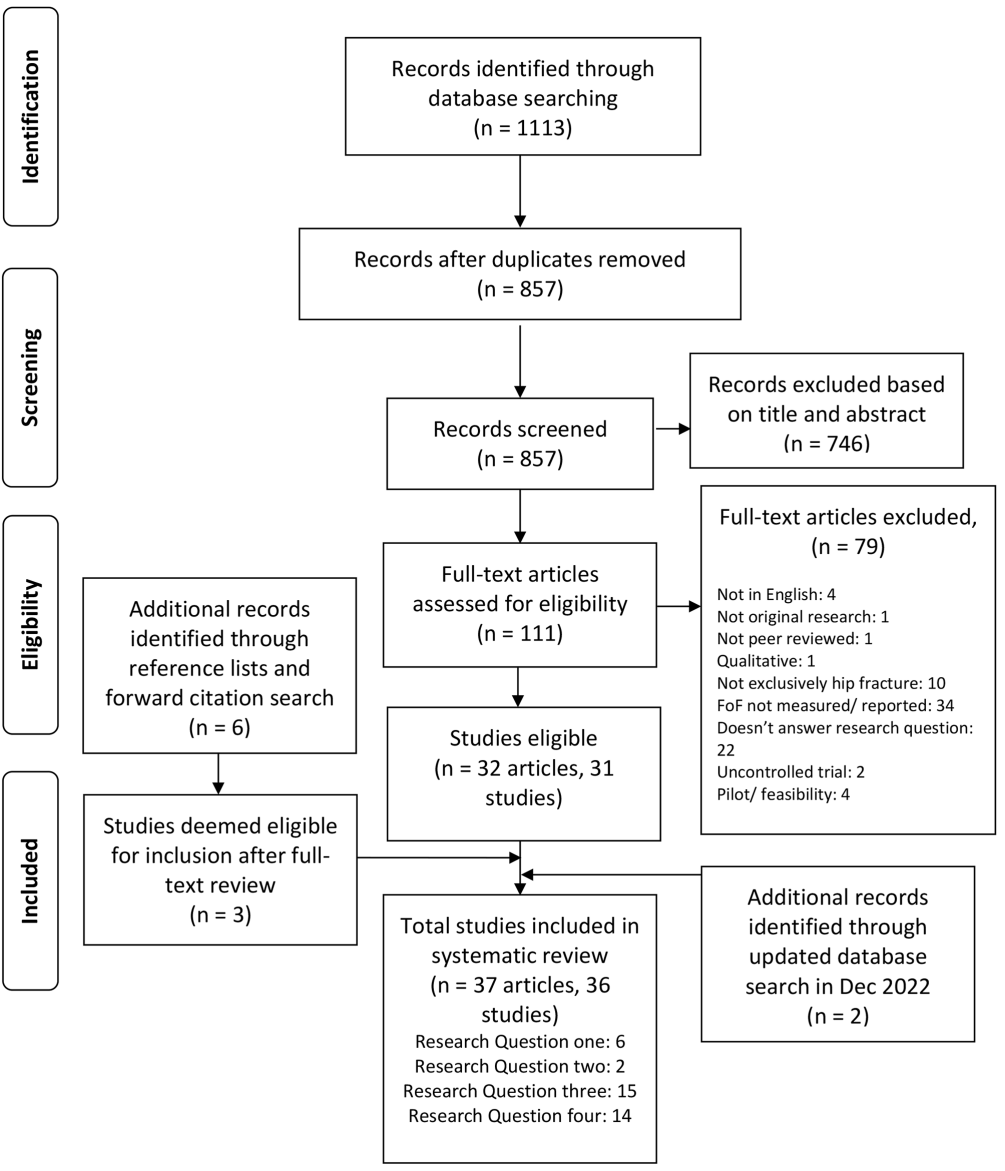
Flow chart showing study screening and selection process

The search in the chosen databases in January 2021 yielded a total of 1113 records. 837 records remained after duplicates were removed. Following screening, 111 records were shortlisted for full-text review based on title and abstract. Finally, 32 articles (31 studies) met criteria and were eligible for inclusion; one of the studies was described in two separate articles [47, 52]. A further six potential studies were identified from reference list and forward citation checks; from these three were eligible for inclusion. Therefore, a total of 35 articles (34 studies) were initially included in this review. In December 2022 the search was updated and a further two studies were identified. Of the final 36 included studies: six answered research question 1, two answered research question 2, fifteen answered research question 3, and fourteen answered research question 4 (note: some studies answered more than one research question). Figure 1 portrays the study screening and selection process.

### Study characteristics

Tables 1, 2, 3 and 4 present the main aims, design, and sample characteristics from included studies for each of the four research questions, respectively. All studies included hip fracture patients (total 5099 participants across studies), usually older than 60 years of age. Female participants made up a greater proportion of the sample consistently across all studies (range 60–100%). Common exclusion criteria seen in most studies were participants with cognitive impairment, need for assistance with mobility pre-fracture, and presence of co-morbidities. The days since hip fracture ranged widely (from within 1 week of hip fracture to 4 years post-fracture) across included studies; but a number of studies did not report this.

**Table 1:** Study design, participant characteristics and data extracted for FoF prevalence studies

Study and Design	Aim	Setting	Sample size (n)	Age (years) Mean ± SD if given	Gender (% female)	Time since hip fracture	FoF measure	RESULT Prevalence (%)
**Bower 2016** [14] *Prospective, longitudinal*	Describe rates of FoF at 4 and 12 weeks post-fracture	8 Hospitals	Start: 299 End: 241	77.2 ± 8.5	74%	Within 1 week of fracture	sFES-I, dichotomised at score of ≥ 11/ 28 (classified as high FoF)	4 weeks: 60.5% 12 weeks: 47.0%
**Jaatinen 2022** [59] *Cross-sectional for FoF data*	Investigate factors associated with post-hip fracture FoF	Hospital	916	Not reported	72%	4–6 months post-fracture	SIQ: (“Do you have a fear of falling?” or “Are you afraid of falling?”)	4–6 months: 49%
**Koeda 2011** [93] *Prospective*	Study effects of FoF on physical function during acute phase	Hospital	Start: 46 End: 40	79.2 ± 6.4	100%	Within 1 week post-operatively	SIQ “Are you currently afraid of, or worried about falling?“	Week 1: 100% Week 4: 50.0%
**Kornfield 2017** [94] *Prospective, longitudinal*	Explore rates and correlates of post-traumatic stress disorder	8 Hospitals	Start: 456 Week 4: 386 Week 12: 352	78.8 ± 8.7	77%	2 days after surgery	SIQ (Item 4 of FFQ) “I am afraid of falling again”	4 weeks: 66.6% 12 weeks: 58.5%
**Ungar 1986** [95] *Prospective*	Not stated	Rehabilitation unit	Start: 72 End: 59	81.0	85%	‘After hospitalisation’, exact timeframe not reported	Not reported	2–6 months: 50.0% 6–12 months: 37.5% 12–15 months: 22.5%
**Visschedijk 2013** [96] *Cross-sectional*	Determine prevalence of FoF	10 post-acute geriatric rehabilitation wards in nursing homes	100	83.1	75%	Within two weeks of fracture	SIQ “Are you afraid of falling”	T1 (mean 21 days): 62.0% T2 (mean 42.2 days): 68.0% T3 (mean 87.7 days): 59.0%

FoF, fear of falling; SD, standard deviation; sFES-I, short falls efficacy scale international; SIQ, single item question; FFQ, fear of falling questionnaire

**Table 2:** Study design and participants characteristics for studies on psychometric properties of FoF instruments

Study and Design	Main aim and Setting	Sample size (n)	Age (years) Mean ± SD	Gender (% female)	Time since hip fracture as reported
**Bower 2015** [20] *Psychometrics testing*	To test the psychometric properties of the FFQ-R (full 15-item version and a shorter 6-item version) *Hospital*	405 (16 for test-retest reliability)	78.0 ± 8.7	75%	Recruited approximately 2 days after surgery Measures taken at 4 weeks
**Visschedijk 2015** [21] *Psychometrics testing*	To test the psychometric properties of the FES-I in hip fracture patients *10 different Skilled Nursing Facilities in Netherlands*	Sample 1			
		100	83.1 ± 8.3	75%	44.5 days٭ (28–63 range) [53]
		Sample 2			
		21	83.2 ± 7.2	90%	3–4 weeks after admission to rehabilitation

FoF, fear of falling; SD, standard deviation; FFQ-R, fear of falling questionnaire revised; FES-I, falls efficacy scale international

٭median

**Table 3:** Study design, participant characteristics and outcome measures for FoF association studies

Study and Design	Main aims	Sample size (n)	Age (years) Mean ± SD	Gender (% female)	Time (days) since hip fracture: Mean ± SD	FoF measure	Physical function or performance measure(s)
**Abel 2020** [55] *Longitudinal*	Explore predictors of change in physical performance	Start: 127 End: 102	84.7 ± 6.5	83%	Recruited within 3 months of fracture Follow-up: 18.5٭ (IQR 14 − 25 days)	sFES-I, FFQ-R	Change in physical performance (Δ in SPPB score)
**Briggs 2018** [61] *Cross-sectional*	Investigate contribution of weight-bearing asymmetry during STS on physical function	31	77.7 ± 10.5	68%	124.7 ± 42.6 (4.1 ± 1.4 months)	ABC	LEM, mPPT, SCT
**Edgren 2013** [23] *Cross-sectional*	Investigate associations between balance confidence, functional balance and physical disability	159	77.4 ± 7.2	73%	620.5 ± 766.5 (1.7 ± 2.1 years)	ABC (Finnish version)	BBS, Physical disability questionnaire
**Ingermarsson 2000** [97] *Cross-sectional*	Investigate the relation between fall-related efficacy and balance	55	82.3 ± 6.8	85%	25.3 ± 13.2 (post-surgery)	Swedish FES, SIQ “Are you afraid of falling?“ with four-point ordinal scale	Sway index on balance platform, FR
**Jaatinen 2022** [59] *Cross-sectional*	Investigate factors associated with post-hip fracture FoF	916	Not reported	72%	4–6 months post fracture	SIQ: (“Do you have a fear of falling?” or “Are you afraid of falling?”)	TUG
**Jellesmark 2012** [62] *Cross sectional*	Investigate the association between FoF and functional ability	33	81.0٭ (65–92 range)	79%	Not reported (recruited 3 months post discharge)	FES-I, mSAFE	FRS, NMS
**Kline Mangione 2007** [58] *Cross-sectional*	Examine relationship of risk factors and impairments on the functional limitation of gait speed	42	79.2 ± 7.6	69%	122.5 ± 58.1 (17.5 ± 8.3 weeks)	ABC	Gait speed on Gait Mat II
**Kneiss 2015** [98] *Cross-sectional*	Examine correlations between vGRF variables and specific clinical variables	29	80.4 ± 7.3	76%	79.1 ± 27.4 (2.6 ± 0.9 months)	ABC	Knee extension strength (involved and uninvolved sides)
**Kronborg 2016** [99] *Cross-sectional data within a Prospective Study*	Measure association between 24-hour upright time and FoF	20	80.0 ± 8.4	78%	6.7 ± 2.4 (after surgery)	sFES-I	Time spent in sit/lie, standing and walking using ActivPal3 accelerometer
**McKee 2002** [54] *Prospective*	Assess if FoF predicts health outcomes after falls	Start: 82 End: 57	80.2 ± 7.3	90%	Recruited 5–8 days after surgery Follow-up 2 months	Single interview question (worry over further falls in next 2 months), FES	FLP
**Oude Voshaar 2006** [18] *Longitudinal study (re-analysis from two RCTs)*	Examine the effect of FoF (at baseline and 6 weeks) on functional outcome at 6 months	Start: 291 End: 187	79.8 ± 8.7	78%	Recruited within 2 weeks post-surgery Follow-up 6 weeks, 3 months and 6 months	mFES	TUG, gait speed, FR, SIP questionnaire
**Portegis 2012** [57] *Cross-sectional*	Examine relationship between performance/ self-report mobility and balance measures	130	77.6 ± 7.2	75%	547.5 ± 730 (1.5 ± 2.0 years)	ABC (Finnish version)	BBS, 10MWT, mTUG, Self-reported mobility questionnaire, maximum voluntary knee extension strength
**Sihvonen 2009** [56] *Cross-sectional*	Examine difference between hip fracture vs. no fracture on balance/ balance confidence	79	75.3 ± 6.7	68%	1542.8 ± 868 (4.2 ± 2.4 years)	ABC	BBS
**Whitehead 2003** [63] *Cross-sectional data within a Prospective Study*	Compare 4 month outcomes of hip fracture patients	73	81.3 ± 6.2	70%	4 months post discharge	FES, ABC	BBS, LHS, Gait speed
**Willems 2017** [64] *Cross-sectional*	Examine the relation between physical activity/ function and FoF	100	83.1 ± 8.3	75%	44.5٭ (28–63 range)	FES-I	Step count using pedometer, POMA

FoF, fear of falling; SD, standard deviation; IQR, interquartile range; sFES-I, short falls efficacy scale international; FFQ-R, fear of falling questionnaire revised; SPPB, short physical performance battery; STS, sit to stand; ABC, activities-specific balance confidence scale; LEM, lower extremity measure; mPPT, modified physical performance test; SCT, stair climb test; BBS, berg balance scale; FES, falls efficacy scale; SIQ, single item question; FR, functional reach test; FES-I, falls efficacy scale international; mSAFE, modified survey of activities and fear of falling; FRS, functional recovery score; NMS, new mobility score; vGRF, vertical ground reaction force; RFD, rate of force development; FLP, functional limitation profile; mFES, modified falls efficacy scale; TUG, timed up and go test; SIP, sickness impact profile; 10MWT, 10 m walk test; mTUG, modified timed up and go test; LHS, London handicap scale; POMA, performance-oriented mobility assessment

٭median

**Table 4:** Study design, participant characteristics, intervention/ control group and follow-up details for FoF intervention studies

Study and Design	Setting	Sample size (n)	Age (years), Mean ± SD	Gender (% female)	Time since hip fracture/ surgery, Mean ± SD unless stated otherwise	Follow-up time-point(s)	Loss to follow-up/ drop-out rate (%)
**EXERCISE BASED**							
**Beckmann 2021** [100] *Parallel-group, pseudo-RCT*	Nursing homes after hospital discharge	IG: Health professional led functional exercise programme in addition to usual care. Up to 4 times daily, 7 days a week for 2 weeks			Not reported (recruited during sub-acute rehabilitation)	2 weeks and 3 months	None
		78	84.8 ± 7.2	81%			
		CG: Usual Care and physiotherapy					
		62	85.5 ± 7.1	81%			
**Taraldsen 2019** [50] *RCT, stratified*	Home, community	IG: 2 exercise sessions (PT led, balance and gait) per week for 10 weeks in addition to usual care			4 months post-surgery	2 and 8 months	21%
		70	84.0 ± 6.6	77%			
		CG: Usual care and rehabilitation					
		73	82.7 ± 5.7	77%			
**van Ooijen 2016** [29] *RCT, parallel group*	Discharge from hospital to a Residential and Rehabilitation Centre	AT: 15 sessions of adaptability treadmill training and 15 sessions of usual physiotherapy over 6 weeks			13٭ (7–65 range) days	4 weeks and 12 months	51%
		24	82.9 ± 6.5	67%			
		CT: 15 sessions of treadmill walking and 15 sessions of usual physiotherapy			13٭ (6–63 range) days		
		23	83.9 ± 5.5	61%			
		CG: 30 sessions of usual physiotherapy			14* (7–79 range) days		
		23	83.3 ± 8.0	91%			
**PSYCHOLOGICALLY BASED**							
**O’Halloran 2016** [92] *RCT*	Participant’s home, community	IG: Motivational Interviewing (1 × 30 min session per week over 8 weeks) in addition to usual care			183 ± 63 days	9 weeks	17%
		13	83.0 ± 4.8	85%			
		CG: Usual care					
		12	82.3 ± 5.7	83%			
**MULTI-COMPONENT (COMBINED EXERCISE AND PSYCHOLOGICAL INTERVENTIONS)**							
**Asplin 2017** [48] *Prospective, controlled, intervention study*	In-patient rehabilitation ward	IG: Psychological component: enhanced OT/PT collaboration, goal setting, supporting patient self-efficacy. Physical component: training kit with instructions, enhanced exercise with protocol, collaboration meetings.			Not reported, but acute, immediately post-operative	Discharge, 1 month	16%
		63	82.0 ± 8.0	75%			
		CG: Standard rehabilitation from OT/ PT					
		63	80.5 ± 7.7	78%			
**Lee 2022** [101] *RCT*	Home, community	IG: 24 sessions plus weekly phone call. Psychological component: motivational counselling, education. Physical component: personalized strength, balance and mobility training. Also: modifications to home environment, education on assistive device use, pressure ulcer care, nutrition management.			55.0 ± 36.3 days (IG) 63.1 ± 26.2 days (CG)	4 and 8 weeks (mid and end of intervention)	28%
		20	78.9 ± 11.7	75%			
		CG: Home exercise instructions using leaflet plus 2 sessions from physiotherapist plus weekly phone call.					
		20	74.3 ± 9.2	75%			
**Pfeiffer 2020** [28] *RCT, extracted data for hip fracture patients only as obtained from lead author*	Recruited from in-patient rehabilitation but seen for intervention approx. 2 months post-discharge	IG: 8 individual sessions incorporating CBT with balance and strength exercise and 4 telephone calls and 1 home visit post-discharge (in addition to usual care), provided by PT who was supervised by a clinical psychologist			Not reported approx. 8 weeks	Before discharge, 3 months after discharge	16%
		42	82.3 ± 6.5	76%			
		CG: Usual rehabilitation for 3 weeks, no further contact after discharge					
		51	82.2 ± 6.6	73%			
**Scheffers-Barnhoorn 2019** [27] *RCT, cluster*	11 Geriatric Rehabilitation (in-patient) units	IG: ‘FIT-HIP’ consisting of CBT elements aimed at reducing FoF (psycho-education, guided exposure to feared activities, cognitive restructuring) integrated with physiotherapy and exercise sessions, provided by PT trained and supported by psychologist			Not reported, but immediate/ acute	Discharge, 3 and 6 months	36%
		39	83.7 ± 7.3	87%			
		CG: Usual multi-disciplinary rehabilitation, including 5–6 physiotherapy sessions per week					
		38	81.3 ± 7.9	71%			
**ACCELERATED/ SUPPORTED DISCHARGE**							
**Crotty 2002** [51] *RCT*	Home after hospital discharge	IG: Accelerated discharge and home-based rehabilitation including initial home visit to address home modifications, then follow-up visits from PT/OT and MDT			Not reported, but immediate/ acute	4 months	None
		34	81.6٭	62%			
		CG: Usual rehabilitation care in hospital					
		32	83.5٭	75%			
**Lockwood 2019** [66] *RCT*	Acute and rehabilitation ward, hospital	IG: Single home visit by OT (participant present on visit) with education, advice, home adaptations, in addition to usual care			Not reported, acute, immediate post-operation	30 days and 6 months	23%
		37	83.4 ± 7.1	76%			
		CG: Usual MDT rehabilitation care					
		40	80.9 ± 7.3	68%			
**Ziden 2008** [52] **and 2010** [47] *RCT*	Home after hospital discharge	IG: Supported discharge (goal setting, motivation and self-efficacy actions, home services, relatives involved, PT/OT accompanied participant home at discharge, follow-up home visits for 3 weeks)			Not reported, but immediate, acute at time of recruitment	1, 6 and 12 months after discharge	9%
		48	81.2 ± 5.9	60%			
		CG: Usual MDT rehabilitation care					
		54	82.5 ± 7.6	78%			
**OTHER**							
**Birks 2003** [67] *RCT*	Community-dwelling	IG: 3 pairs of hip protectors issued and general advice leaflet on how to reduce fracture risk			Not reported, any time, no restrictions	6 weeks and 6 months	24%
		182	80.8 ± 6.0	87%			
		CG: Leaflet only					
		184	80.2 ± 5.7	88%			
**Ko 2019** [68] *Quasi-experimental, pre-test post-test design, with non-equivalent control group*	Orthopaedic ward, hospital	IG: Individualised transitional care programme: nurse led, primarily educational programme via booklets, observation, demonstration and therapeutic communication (included goal setting, emotional support, positive reinforcement), 6 times for 2 weeks			Not reported, but immediate, acute	1–2 days before discharge	8%
		18	75.5 ± 3.7	78%			
		CG: Usual post-operative care plus booklets					
		16	77.9 ± 5.4	81%			
**Peichl 2005** [69] *RCT, parallel group*	Rehabilitation ward, hospital	IG: 200IU salmon calcitonin nasal spray twice daily for 12 months in addition to 1000 mg calcium and 880IU vitamin D daily			Not reported, but acute, post-operative	12 months	35%
		37	78.9 ± 6.3	100%			
		CG: 1000 mg calcium and 880IU vitamin D daily for 12 months					
		38	76.9 ± 3.9	100%			

FoF, fear of falling; SD, standard deviation; RCT, randomised controlled trial; IG, intervention group; CG, control group; PT, physiotherapist; OT, occupational therapist; CBT, cognitive behavioural therapy; MDT, multi-disciplinary team

٭ median

### Risk of bias in studies

All studies were critically appraised for their methodological quality using the chosen tools for each research question. The main appraisal findings are provided in tables in supplementary file 2.

### Prevalence

**Fig. 2:**
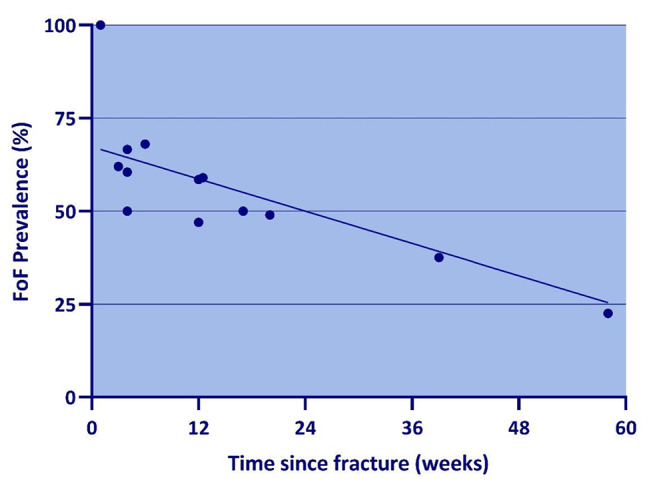
Prevalence of FoF among hip fracture patients

Of the six included studies that measured and reported FoF prevalence in hip fracture patients, four were prospective cohort studies and two were cross-sectional in design. The study characteristics and main prevalence data extracted from these studies are presented in Table 1. As outlined in the table, each study measured FoF prevalence using a different tool and at varying time points after hip fracture. At 1–4 weeks post-fracture, FoF prevalence ranged between 50 and 100%, at ~ 12 weeks the range was between 47 and 59% and for the 12–58 week period it ranged between 23 and 50%. The scatter graph (Fig. 2) shows that FoF prevalence reduced as the time since hip fracture increased. Most studies had at least a moderate risk of bias on the appraisal tool. The main source of bias was use of convenience samples. Also, most studies used a single item questionnaire (SIQ) to measure FoF prevalence, but the reliability and validity of such an approach is not yet clear [8].

### Instrument psychometrics

Two eligible studies were found that measured the psychometric properties of FoF instruments in the hip fracture population. Descriptions of these studies are presented in Table 2.

Results of psychometric testing are shown in Table 5. Bower et al. [20] measured the psychometric properties of both a 15-item and a shorter 6-item version of the ‘Fear of falling questionnaire revised’ (FFQ-R). They found that the 15-item and 6-item versions demonstrated acceptable and good internal consistency as well as excellent and good test-retest reliability, respectively. They also showed adequate construct validity as both correlated with the Short Falls Efficacy Scale – International and showed divergence from scores for depression and negative affect.

**Table 5:** Results of psychometric properties of the FFQ-R (15 and 6-item) and the FES-I

	Bower 2015 [20]		Visschedijk 2015 [21]
**Psychometric property**	**15-item FFQ-R**	**6-item FFQ-R**	**FES-I**
Internal Consistency	Acceptable, Cronbach’s alpha 0.76, [0.73, 0.80 95%CI]	Good, Cronbach’s alpha 0.80, [0.77, 0.83 95% CI]	Excellent, Cronbach’s alpha = 0.94
Reliability	Test-retest Reliability – Excellent, ICC = 0.93, [0.85, 1.0 95%CI]	Test-retest Reliability – Good, ICC = 0.82, [0.65, 0.99 95%CI]	Inter-rater Reliability – Moderate, ICC = 0.72, [0.52, 0.87 95%CI]
Measurement Error	-	-	Substantial: SEM = 6.4 ; SDC = 17.7
Construct Validity	Convergent Validity – Adequate, hypothesis confirmed, Moderate correlation with sFES-I (r = 0.43) Divergent Validity – hypotheses confirmed, Weak correlation with MADRS (r = 0.25); Weak correlation with negative PANAS (r = 0.32)	Convergent Validity – Adequate, hypothesis confirmed, Moderate correlation with sFES-I (r = 0.42) Divergent Validity – hypotheses confirmed, Weak correlation with MADRS (r = 0.26); Weak correlation with negative PANAS (r = 0.34)	Construct validity – Questionable, 4 out of 11 hypotheses confirmed, strongest correlation with single item FoF instrument (r = 0.68). The FES-I correlated more closely with physical function compared to psychological scales.
Structural Validity	Using factor analysis found a 4 factor solution (threat, future expectancy, coping and harm)	Using factor analysis found a 2 factor solution (threat and harm)	Factor analysis: no item had a factor loading of ≤ 0.50, Strong evidence for uni-dimensionality of FES-I
Floor and Ceiling effects	-	-	Floor and Ceiling effects – none, 0% participants had maximum score and 1% had minimum score

FFQ-R, fear of falling questionnaire revised; FES-I, falls efficacy scale international; CI, confidence interval; ICC, intraclass correlation co-efficient; SEM, standard error of measurement; SDC, smallest detectable change; sFES-I, short falls efficacy scale international; MADRS, Montgomery asberg depression rating scale; PANAS, positive and negative affect schedule; r, Pearson or Spearman’s correlation coefficient

Visschedijk et al. [21] investigated the psychometric properties of the Falls Efficacy Scale – International (FES-I) in hip fracture patients. Table 5 shows that the FES-I had excellent internal consistency and moderate inter-rater reliability. The standard error of measurement and the smallest detectable change were both high, suggesting that the scale has substantial measurement error, as acknowledged by the authors. As part of construct validity testing, only four out of 11 hypotheses were confirmed with the FES-I score found to be more closely correlated to measures of physical and functional performance (e.g. performance oriented mobility assessment and timed up and go test) than psychological constructs relating to fear, depression or anxiety. Thus, the construct validity testing suggests that this scale may not capture the emotional aspects of FoF but is better suited to measuring the functional performance aspects. The FES-I did not demonstrate any floor or ceiling effects.

The quality assessment found that internal consistency and construct validity were appropriately tested. Visschedijk et al. [21] did not score well for measurement error testing and concerns were identified for structural validity testing but the reason was minor (they did not report the rotation method for factor analysis). Both studies also scored poorly for test-retest reliability methods because the interval between tests was short, but this is probably unavoidable in a rehabilitation setting where clinical change is likely over a longer time period. Overall, the studies were well conducted.

#### Associations with measures of physical function or performance

Fifteen studies were included to answer research question 3 (Table 3). Most studies were cross-sectional, but three were prospective [18, 54, 55]. Results were grouped according to the category of functional performance that was measured.

- Balance.

Six studies investigated the association between FoF and balance measured using outcome measures like the Berg balance scale and Functional reach test (Table 6). All studies that assessed the significance of these relationships reported at least one significant association between FoF and balance. The only study which did not assess significance reported a strong positive correlation [56]. Overall these studies suggest that FoF is consistently associated with poorer balance.

**Table 6:** Results of studies assessing associations between fear of falling and physical function

Study	FoF measure	Function measure	Result (correlation/OR (CI)/Std β)	Effect size / Interpretation
**Balance**				
Edgren 2013 [23]	ABC (Finnish)	BBS	r = 0.69*	Large
Ingemarsson 2000 [97]	FES (Swedish)	Sway on Balance Platform	r = -0.42*	Medium
	SIQ	Sway on Balance Platform	r = 0.34*	Medium
	FES (Swedish)	FR	r = 0.53*	Large
	SIQ	FR	r = -0.20ns	Small
Oude Voshaar 2006 [18]	mFES at baseline	FR at 6mo	OR = 1.06ns (0.92–1.21), ES = 0.03	Negligible, ns
	mFES at 6wks	FR at 6mo	OR = 1.32* (1.08–1.60), ES = 0.15	Negligible
Portegis 2012 [57]	ABC (Finnish)	BBS	r = 0.72*	Large
	ABC score < 85	BBS	**OR 12.60 (5.30–29.80)**, ES = 1.40	Large
Sihvonen 2009 [56]	mABC	BBS	r = 0.74 (significance not stated)	Large
Whitehead 2003 [63]	FES	BBS	r = 0.55*	Large
	mABC	BBS	r = 0.77*	Large
**Gait Speed**				
Kline Mangione 2007 [58]	ABC	Gait speed	r = 0.61*	Large
			r² = 0.035*, Std β = 0.222	BC explained 3.5% of the variance
Kronborg 2016 [99]	sFES-I	10MWT	r = -0.50*	Large
Oude Voshaar 2006 [18]	mFES at baseline	Gait speed at 6mo	OR = 0.93ns (0.82–1.04), ES = 0.04	Negligible, ns
	mFES at 6wks	Gait speed at 6mo	OR = 0.73* (0.62–0.86), ES = 0.17	Negligible
Portegis 2012 [57]	ABC (Finnish)	10MWT	r = 0.51*	Large
	ABC score < 85	10MWT	OR 6.30* (2.60–15.00), ES = 1.02	Large
Whitehead 2003 [63]	FES	Gait speed	r = 0.38*	Medium
**Composite Function**				
Abel 2020 [55]	sFES-I	∆ SPPB at f/up (< 1mo)	ns, not entered into regression model	-
	FFQ-R	∆ SPPB at f/up (< 1mo)	Std β = -0.279*	Medium
Briggs 2018 [61]	ABC	mPPT	r = 0.77*	Large
			Std β = 0.61*, part. r = 0.32	BC explained 10.4% of the variance
	ABC	SCT	r = -0.65*	Large
			Std β = -0.37*, *, part. r = -0.20	BC explained 3.8% of the variance
Jaatinen 2022 [59]	SIQ	TUG	OR (moderately abnormal) = 1.46 (1.08–1.97)*, ES = 0.21 OR (markedly abnormal) = 2.45 (1.36–4.42)*, ES = 0.50 Multivariate adjusted: OR (moderately abnormal) = 1.39 (0.97–1.98), ES = 0.18 OR (markedly abnormal) = 3.14 (1.49–6.63)*, ES = 0.63	Small to Medium
Kronborg 2016 [99]	sFES-I	TUG	r = 0.54*	Large
Oude Voshaar 2006 [18]	mFES at baseline	TUG at 6mo	OR = 0.89* (0.80–0.99), ES = 0.06	Negligible
	mFES at 6wks	TUG at 6mo	OR = 0.75* (0.64–0.88), ES = 0.16	Negligible
Portegis 2012 [57]	ABC (Finnish)	mTUG	r = -0.56*	Large
	ABC score < 85	mTUG	OR 7.30* (3.00–17.80), ES = 1.10	Large
Willems 2017 [64]	FES-I	POMA	r = 0.43*	Medium
**Self-reported function**				
Edgren 2013 [23]	ABC (Finnish)	Physical Disability questionnaire	IRR 0.99*, (0.98–0.99), p < 0.001	For every 10 point increase in BC, disability score reduced by 10%
Jellesmark 2012 [62]	FES-I	FRS	r = -0.78*	Large
	mSAFE	FRS	r = -0.80*	Large
	FES-I	NMS	r = -0.67*	Large
	mSAFE	NMS	r = -0.74*	Large
Mckee 2002 [54]	FES at baseline	FLP at 2mo	r = -0.37* Std β = − .016ns, r² = 0.05	Medium FE explained 5% variance, ns
	SIQ at baseline	FLP at 2mo	r = 0.18ns	Small
Oude Voshaar 2006 [18]	mFES at baseline	SIP mobility at 6mo	OR = 0.92 (0.83–1.02), *p =* 0.11, ES = 0.04	Negligible
	mFES at 6wks	SIP mobility at 6mo	OR = 0.70* (0.60–0.81), *p <* 0.001, ES = 0.20	Small
	mFES at baseline	SIP activity at 6mo	OR = 0.90* (0.81-1.00), p = 0.05, ES = 0.06	Negligible
	mFES at 6wks	SIP activity at 6mo	OR = 0.71* (0.61–0.82), p < 0.001, ES = 0.19	Negligible
Portegis 2012 [57]	ABC (Finnish)	Ability to walk outdoors	r = -0.54*	Large
	ABC score < 85	Ability to walk outdoors	**OR 18.7 (6.00–58.00)**, ES = 1.62	Large
	ABC (Finnish)	Self-reported stair climb	r = -0.57*	Large
	ABC score < 85	Self-reported stair climb	**OR 11.7 (4.60–29.90)**, ES = 1.36	Large
Whitehead 2003 [63]	FES	LHS	r = 0.62*	Large
	ABC	LHS	r = 0.80*	Large
**Physical activity**				
Kronborg 2016 [99]	sFES-I	Time spent upright (accelerometer)	r = -0.48*	Medium
Willems 2017 [64]	FES-I	Step count (pedometer)	r = 0.34*	Medium
			OR = 0.94* (0.89–0.99), ES = 0.03	Negligible
			ns, statistic not reported	-
**Muscle Strength**				
Kneiss 2015 [98]	ABC	Knee extension strength, involved; uninvolved side	r = 0.55;* r = 0.52*	Large
Portegis 2012 [57]	ABC (Finnish)	Knee extension strength	r = 0.40*	Medium

FoF, fear of falling; OR, odds ratio; CI, confidence interval; Std β, standardized beta coefficient; ABC, activities-specific balance confidence scale; BBS, berg balance scale; FES, falls efficacy scale; SIQ, single item question; FR, functional reach test; ns, non-significant; wks, weeks, mo, months; mFES, modified falls efficacy scale; ES, effect size; mABC, modified activities-specific balance confidence scale; sFES-I, short falls efficacy scale international; 10MWT, 10 m walk test; FFQ-R, fear of falling questionnaire revised; ∆ SPPB, change in short physical performance battery; mPPT, modified physical performance test; SCT, stair climb test; TUG, timed up and go test; mTUG, modified timed up and go test; FES-I, falls efficacy scale international; POMA, performance-oriented mobility assessment; IRR, incident rate ratio; ADL, activities of daily living; IADL, instrumental activities of daily living; mSAFE, modified survey of activities and fear of falling; FRS, functional recovery score; NMS, new mobility score; SIQ, single item question; FLP, functional limitation profile; SIP, sickness impact profile; LHS, London handicap scale

**statistically significant**; ns statistically non-significant

(b) Gait speed.

Five studies reported findings on gait speed and all reported significant associations with FoF (Table 6). The bivariate correlation coefficients indicated the association had a medium to large effect size. One study also found a strong association after controlling for potential confounders [57], and another found a prospective association [18] when gait speed was assessed 6 months later. In contrast, Kline Mangione et al. [58] showed that balance confidence explained only 3.5% variance in gait speed, although this was still statistically significant. Overall, these findings suggest that FoF is associated with slower gait speed.

(c) Composite physical performance measures.

Seven studies reported on associations between FoF and outcome measures that tested participants on more than one aspect of physical performance (such as a combination of mobility and balance tasks e.g. Short Physical Performance Battery and Performance Oriented Mobility Assessment). All studies that performed correlations found a significant, small to large correlation between FoF and function (Table 6). Three cross-sectional studies found associations remained significant after controlling for covariates, at least for some analyses [59–61]. Two prospective studies found that FoF does predict future functional performance when assessed a few weeks to 6 months later [18, 55]. Overall, these findings suggest that FoF is associated with poorer performance in composite physical performance measures.

(d) Self-reported function.

Six studies used self-report questionnaires to measure function (Table 6). All four cross-sectional studies reported significant associations, two after controlling for covariates [23, 57, 62, 63]. Of the two prospective studies, one found that FoF did not predict later function [54] and the other found a small or negligible effect size [18]. Therefore, FoF is likely correlated with self-reported function when assessed concurrently, but its ability to predict future self-reported function may be limited.

(e) Physical activity.

Two studies assessed associations between FoF and activity levels measured with an accelerometer or pedometer (Table 6). Correlations were significant with a medium effect size. However this relationship did not remain significant after controlling for covariates [64], and there were no prospective studies in this category. Thus, the relationship between FoF and physical activity measures remains uncertain, with limited evidence that FoF is related to decreased physical activity.

(f) Muscle strength.

Two studies reported on associations between FoF and quadriceps strength (Table 6). They demonstrated that balance confidence was associated with quadriceps strength, with medium to large correlation coefficients. No studies attempted to control for potential confounding factors and no prospective study measured this association. Thus, there is limited evidence that higher FoF may be related to reduced quadriceps strength.

The quality appraisal of these studies showed that studies scored well for having clear aims, appropriate study designs, ethical conduct and using validated and reliable measures. However few studies controlled for confounding variables like age, co-morbidities, pre-fracture function and falls history in their analyses. Another source of bias was participant selection; most studies did not ensure that the sample was representative e.g. excluding participants with cognitive impairment or co-morbidities. Additionally, small sample sizes and low response rates mean the studies are unlikely broadly representative of the hip fracture population.

### Interventions

Fourteen studies (fifteen articles) were included that assessed effects of an intervention on FoF. Most studies included FoF as a secondary measure; only five studies had a primary aim of reducing FoF [27–29, 51, 52]. One study had a quasi-experimental pre-test post-test design [65] and one was a non-randomised controlled intervention study [48], the remaining were RCTs. The main characteristics and data extracted from these studies are given in Table 4. The interventions were loosely categorized as follows: exercise based, psychologically based, multi-component (commonly combining exercise and psychological intervention strategies), accelerated or supported discharge, and other. The main results are provided in Table 7. On the quality appraisal tool, four studies achieved ‘some concern’ and the remaining 10 studies achieved ‘high risk of bias’. Common sources of bias were inadequate allocation concealment and/or baseline differences between groups, non-blinded participants and clinicians as well as lacking an intention-to-treat analysis. Also, while most studies undertook a power calculation to justify their sample size, few explicitly calculated this in relation to their FoF measure.

**Table 7:** Results of all intervention studies

Study		FoF outcome measure used		Measurement time point		Result (mean ± SD) unless otherwise stated		Effect Size (Cohen’s D)		Interpretation	
**EXERCISE BASED**											
Beckmann 2021 [100]		FES-I		2 weeks		IG: 38.0 ± 12.8ns CG: 38.6 ± 14.3		0.03		Negligible effect	
				3 months		IG: 29.3 ± 11.5ns CG: 31.6 ± 13.2		0.13		Negligible effect	
Taraldsen 2019 [50]		sFES-I		2 months (adjusted for baseline)		Between group difference: mean = -0.2 (-1.3, 0.9 95% CI)ns		Unable to calculate		No significant difference between groups	
				8 months (adjusted for baseline)		Between group difference: mean = 0.1 (-1.3, 1.3 95% CI)ns		Unable to calculate		No significant difference between groups	
Van Ooijen 2016 [29]		FES-I		Post-intervention		n² = 0.057ns		0.11		Negligible effect	
				4 weeks		n² = 0.016ns		0.03		Negligible effect	
				12 months		n² = 0.045ns		0.09		Negligible effect	
**PSYCHOLOGICALLY BASED**											
O’Halloran 2016 [92]		mFES		9 weeks (without adjusting for baseline)		IG: 8.4 ± 2.1* **CG: 6.7 ± 2.0**		0.59		Medium effect, FoF score improved in IG more than the CG	
				9 weeks (adjusted for baseline, week 9 minus week 0)		IG: 0.5 ± 0.8* **CG: -0.4 ± 1.0**		0.70		Medium effect	
**MULTI-COMPONENT (COMBINED EXERCISE AND PSYCHOLOGICAL INTERVENTIONS)**											
Asplin 2017 [48]		FES Swedish		Discharge		IG: median 73 (7–125 range)ns CG: median 73 (18–130 range)		-0.10		Negligible effect	
				1 month		IG: median 89 (31–130 range)ns CG: median 90 (16–130 range)		0.09		Negligible effect	
Lee 2022 [101]		FES Korean		4 weeks		IG: 41.6 ± 27.1 CG: 35.3 ± 19.8		0.19		Negligible effect	
				8 weeks		IG: 33.9 ± 26.5 CG: 30.5 ± 15.1		0.11		Negligible effect	
Pfeiffer 2020 [28]		sFES-I		Discharge at end of rehab		IG: 12.63 ± 4.14ns CG: 12.50 ± 4.02		-0.02		Negligible effect	
		PAMF		Discharge at end of rehab		IG: 12.80 ± 2.87ns CG: 12.70 ± 2.29		0.03		Negligible effect	
		sFES-I		3 months since discharge (1 month post intervention)		IG: 11.40 ± 4.94* **CG: 12.80 ± 4.66**		0.21		Small, sFES-I scores improved more in the IG than the CG	
		PAMF		3 months since discharge (1 month post intervention)		IG: 13.30 ± 2.63* **CG: 12.80 ± 2.43**		0.14		PAMF scores improved more in the IG than the CG but negligible effect size	
Scheffers-Barnhoorn 2019 [27]		FES-I		Discharge		IG: 32.8 ± 11.0ns CG: 27.0 ± 8.2		-0.42		Small Effect, At discharge the IG had more FoF than the CG	
				3 month follow-up		IG: 35.1 ± 13.9ns CG: 36.6 ± 12.4		0.08		Negligible Effect	
				6 month follow-up		IG: 36.5 ± 12.1ns CG: 36.5 ± 11.9		0		No Effect	
**ACCELERATED/ SUPPORTED DISCHARGE**											
Crotty 2002 [51]		FES		4 months		IG: 90.5 median, 80.5 (25th percentile), 98.0 (75th percentile)* **CG: 79.5 median, 40.0 (25th percentile), 92.5 (75th percentile)**		Unable to calculate from data provided		IG had a significant improvement in FoF scores compared to CG at 4 months	
		ABC		4 months		IG: 61.3 median, 45.5 (25th percentile), 75.2 (75th percentile)ns CG: 53.3 median, 26.8 (25th percentile), 74.6 (75th percentile)		Unable to calculate from data provided		IG had a slightly better ABC median score than CG at 4 months but not significant	
Lockwood 2019 [66]		FES-I		30 days		IG: 35.1 ± 11.2ns CG: 32.6 ± 13.6		-0.14		Negligible effect	
				6 months		IG: 26.8 ± 8.0ns CG: 28.0 ± 13.1		0.08		Negligible effect	
Ziden 2008 [52] and 2010 [47]		FES Swedish (higher score means higher confidence)		1 month		IG: 117.4 ± 12.0* **CG: 85.5 ± 30.5**		0.97		Large, FOF scores improved in the IG significantly more than the CG at 1 month	
				6 months		IG: 128 median, 20 (min), 160 (max)* **CG: 105 median, 7 (min), 130 (max)**		0.48		Small, FOF scores improved in the IG significantly more than the CG at 6 months	
				12 months		IG: 128 median, 61 (min), 130 (max)* **CG: 102 median, 13 (min), 130 (max)**		0.73		Medium, FOF scores improved in the IG significantly more than the CG at 12 months	
**OTHER**											
Birks 2003 [67]		FoF 6 point Likert scale		6 weeks		IG: 1.73 ± 1.83 (significance not stated) CG: 1.75 ± 1.91		0.01		Negligible difference	
				6 months		IG: 2.59 ± 1.54 (significance not stated) CG: 2.78 ± 1.64		0.08		Negligible difference	
Ko 2019 [68]		FES Tinetti 10 item		1–2 days before discharge, pre-test post-test design		IG: 23.83 ± 29.35* **CG: 36.19 ± 26.86**		0.31		Small, FoF scores improved in the IG more than the CG	
Peichl 2005 [69]		FES Tinetti 14 item		12 months		IG: 3.28 ± 1.24* **CG: 2.29 ± 1.08**		0.60		Medium, FES scores improved in the IG more than the CG	

FoF, fear of falling; Y, yes; N, no; SD, standard deviation; FES-I, falls efficacy scale international; IG, intervention group; CG, control group; sFES-I, short falls efficacy scale international; CI, confidence interval; n², partial eta squared effect size; mFES, modified falls efficacy scale; FES, falls efficacy scale; PAMF, perceived ability to manage falls scale; ABC, activities-specific balance confidence scale

* statistically significant; ns statistically non-significant

Three RCTs, with a total of 353 participants, investigated the effect of exercise based interventions such as balance and gait exercises (see Table 4). None of these studies found a significantly greater improvement in FoF compared to control groups, which all included usual care physiotherapy rehabilitation. As described in Table 4, the frequency, duration and type of exercise varied between the studies.

Only one study used solely a psychologically based intervention (8-week motivational interviewing intervention); it showed a statistically significant improvement in FoF with a medium effect size compared to usual care.

Four studies (total sample size of 336 participants) utilised a combination of exercise based (such as strength, balance and/or mobility training) and psychological interventions (such as cognitive behavioural therapy, motivational counselling and goal setting), consisting of multiple components. Only one of these studies [28] found a statistically significant improvement in FoF measures compared to a control group. This improvement was only seen at follow-up (1 month post-intervention) but not immediately post-intervention and the effect size was small or negligible.

Three RCTs looked at accelerated or early supported discharge compared to usual rehabilitative care, with a total of 245 participants. Two RCTs [51, 52] performed home based rehabilitation along with accelerated/ supported discharge; both reported a statistically significant improvement in FoF compared to usual care. The third RCT [66] provided a single pre-discharge home visit as its main intervention (without any additional home based rehabilitation); this study did not show improvement in FoF.

Lastly, three studies included in our review utilised interventions that did not fit within the preceding categories, so were categorised as ‘other’. Birks et al. [67] assessed the use of hip protectors and did not find a statistically significant result. Ko et al. [68] investigated a nurse led individualised programme consisting of education such as fall prevention, and emotional support to minimise functional decline. They reported a statistically significant improvement in the intervention group compared to the control but the effect size was small. Peichl et al. [69] investigated the effect of a salmon calcitonin spray (administered for one year) on bone density and fracture rate and reported a statistically significant result for FoF improvement with a medium effect size.

## Discussion

### Prevalence

This systematic review found that FoF prevalence ranged between 50 and 100% at 1–4 weeks, 47 to 59% at around 12 weeks and 23 to 50% for the period 12–58 weeks post hip fracture. Thus, FoF is extremely common, especially early after hip fracture. This is the first systematic review to report FoF prevalence estimates after hip fracture; a previous systematic review [15] did not find any studies that adequately reported this. The findings show a trend of decreasing FoF prevalence as time passes since hip fracture. Intuitively, this makes sense because it can be expected that an individual’s FoF would improve as they make progress with their mobility in the later stages of their rehabilitation. These findings highlight the need for clinicians to assess for FoF, particularly in the early rehabilitation phase. Using a validated measure such as the FES-I could provide useful information about which particular activities or tasks the patient fears falling in the most which could help tailor therapy sessions to address FoF during those specific tasks.

### Instrument psychometrics

This review identified three scales that have been assessed for use in the hip fracture population: The FFQ-R (15-item version), the FFQ-R (6-item version) and the FES-I [20, 70]. All showed adequate reliability and factor structure. Both versions of the FFQ-R showed good validity compared to other instruments, though not surprisingly the FES-I (where items focus on efficacy to perform functional tasks) showed better validity for measuring the functional components of FoF rather than tapping into the emotional components.

Our findings are consistent with prior research. The FES-I demonstrated excellent internal consistency and reliability in older adults [71] and in geriatric patients with or without cognitive impairment [72]. It can also be used for older adults of different cultural backgrounds [73] and cut-off scores have been recommended to indicate whether there is a low, moderate or high concern for falling [71]. The FFQ-R findings were consistent with that for the original FFQ which also had acceptable reliability, validity and factor structure [74]. One advantage of the FFQ-R is that it was revised specifically for the hip fracture group and measures fear more globally instead of measuring self-efficacy during specific functional tasks [20].

### Associations with measures of physical function or performance

This review demonstrated consistent associations between FoF and physical function. Greater FoF was associated with poorer balance, strength, physical performance and self-reported function, slower gait speed and reduced physical activity. Some of these associations remained after controlling for covariates, or demonstrated significant longitudinal associations in prospective studies. This relationship between FoF and physical function is consistent with findings from the general older adults population, where FoF is also consistently related to poorer function and predicts future falls [12, 75].

The association between FoF and physical function may be causal, although it is not possible to determine from the included studies. For example, it may be that higher FoF leads to greater disability through sustained fear and avoidance of functional activities. Alternatively, having poor physical function may lead an individual to be more fearful of falling in light of their limited abilities. Finally, a third underlying variable such as frailty, depression or age could explain the association. Few studies were either prospective or controlled for covariates and those that did, demonstrated mixed or weaker associations between FoF and function, suggesting that a direct causal relationship may not exist. However, it seems likely that a vicious cycle of poorer function and FoF may reinforce each other. Interestingly one study found that elevated FoF at 6 weeks post-fracture was a better predictor of later function than FoF at baseline [18], suggesting that those who continue to have elevated FoF after the immediate rehabilitation phase may be at greatest risk of poor function and, therefore, it may be worth targeting FoF early in the rehabilitation process.

### Interventions

A large majority of studies in this review did not find improvements in FoF as an outcome of their chosen interventions. However, most studies only included FoF as a secondary measure. These studies could be underpowered as their sample size was not calculated based on FoF as the primary outcome measure. Furthermore, many of the included studies were considered high risk of bias, making it difficult to draw any strong conclusions regarding interventions to address FoF after hip fracture.

The three studies investigating exercise based interventions did not show improvement in FoF in hip fracture patients. Their control groups did receive usual care and physiotherapy, which typically included some exercise, because ethically, hip fracture patients cannot be denied usual care. Therefore, the dose of exercise provided to the intervention group may not have been sufficiently different to the control group to clearly affect outcomes, including FoF.

Multi-component interventions that combined exercise with psychological interventions (e.g. cognitive behavioural therapy or CBT) also did not show any effect in reducing FoF after hip fracture. Theoretically, a combination of physical and psychological measures should improve FoF; the psychological component empowers the patient with skills to overcome their fear, while the physical component helps improve falls efficacy/ balance confidence by improving strength and balance [25]. In addition, engaging in exercise without catastrophic consequences (i.e. falling) may disconfirm fears that exercise is dangerous and operate as an exposure therapy [76]. Multi-component interventions have shown success in reducing FoF [77, 78] and improving balance confidence [24], in community-dwelling older adults. An important difference could be the setting where these interventions took place; it may be difficult for trials to show a significant improvement in the early stages of hip fracture rehabilitation compared to that in the community. Also, FoF may be more prevalent in the early stages after hip fracture but decreases over time. Thus, it may make it difficult to see a difference between the intervention and control groups if both groups experience a natural reduction in FoF anyway (i.e. as part of natural history). Bower et al. [14] make a similar point, suggesting that high FoF early after hip fracture could be transient and adaptive, but persistent high FoF three months post-fracture could be maladaptive. Therefore, interventions may show a stronger effect on FoF in patients that continue to have residual FoF later on (such as 6–12 weeks post-fracture) compared to early post-fracture.

Accelerated or supported discharge based interventions involving home modifications, advice and education showed mixed results. The studies that added a goal-oriented and tailored home rehabilitation programme provided by therapists did show some improvement in FoF compared to the study with home visits only. However, the effect size varied and the results of one study [47] in particular were biased by methodological flaws that may have resulted in an inflation of the effect in favour of the intervention group. A recent meta-analysis [79] reviewed three RCTs on community-based outdoor mobility interventions on falls efficacy after hip fracture and reported a small increase in falls efficacy; however, upon removing the findings of Ziden et al. [47] due to heterogeneity, they reported that outdoor mobility interventions did not make a difference to falls efficacy.

### Limitations of the existing research on FoF after hip fracture

As a whole, there were some important issues in the quality of the literature reviewed. Firstly, many studies had selection bias as they excluded participants with cognitive impairment, pre-fracture mobility issues or major co-morbidities. Thus, the findings from this review may not be generalisable to all hip fracture patients. Also, FoF may be a greater issue in cognitively impaired patients [80], which has not been studied well in the current literature. Secondly, female participants made up a resounding majority in all studies included in this review. While hip fracture does occur in females more than males (66–69% of patients included in the Australian and New Zealand Hip Fracture Registry 2021 report [81] were female), the average across all studies included in our review was higher at 78%, with some studies including 100% females. It could be that more females consented to participate which may be a potential source of bias in these studies and affects the generalisation of findings to males. Women experience greater levels of anxiety than men and FoF may present differently in females compared to males [82]. Future research could investigate gender differences in the presentation and treatment of FoF. Thirdly, many studies did not clearly report the time since hip fracture. This makes it difficult to appropriately interpret and draw implications from their results as their findings cannot be linked adequately to the participants’ stage of rehabilitation. Lastly, there may still be some lack of clarity about the fall-related psychological construct being measured as different studies and tools emphasize different aspects of FoF such as falls efficacy, balance confidence or fear itself. We suggest that future research focusses on clarifying the construct of FoF and better understanding the relationships between these three components.

### Future research directions

There is a need for more studies to add to and consolidate the evidence base about FoF prevalence in the very early days after hip fracture. Likewise, future prospective studies need to evaluate FoF prevalence over a longer follow-up period (of 1 year and more) in the same participants to investigate how FoF changes as time lapses well beyond the acute hip fracture stage. This will help elucidate whether FoF continues to be an issue once patients have transitioned back into the community. Additionally, as discussed by Bower et al. (2016), high FoF early after hip fracture (e.g. in the first month) may be adaptive (or even protective), however, high FoF much later post fracture (e.g. three months or more) may be maladaptive. This nature of FoF over a period of time post hip fracture needs further investigation. It would be beneficial to include more representative populations (e.g. based on national hip fracture registries) and validated and reliable tools such as the FES-I to measure FoF prevalence rather than a SIQ which has unknown and potentially limited psychometric properties.

Future research should better investigate measurement error and sensitivity to change for all scales, as this was not assessed for the FFQ-R and was poor for the FES-I in the hip fracture population. There are additional FoF scales which are common in clinical practice that have not been investigated in the hip fracture population to determine reliability and validity and this should also be a focus of future studies.

There is still a need for research on effective targeted interventions that can address FoF post hip fracture. One intervention that has not yet received any attention is that of graded exposure therapy. Graded exposure is a common and effective treatment strategy for anxiety disorders as well as pain-related fear and anxiety [83–85]. It has also been used by physiotherapists to address fear avoidance behaviours seen in low back pain patients, with some success [86–88]. In light of the fear avoidance behaviours linked to FoF [89], this intervention has the potential to be similarly effective in addressing FoF after hip fracture. In the FoF context, this could be implemented by graded exposure to the feared activity or task. Given its success in treating other anxiety and fear based disorders, including fear of movement, we recommend investigating this intervention in the hip fracture population. Similarly, no study has investigated solely CBT as an intervention, and further research is needed to establish its effect on FoF after hip fracture. We acknowledge that such therapies may be challenging in frailer patients or those with significant cognitive issues.

The intervention of ‘tai chi’ has also shown positive effect on FoF in older adults [90, 91]. While it may be physically difficult for patients to perform tai chi in the acute stages after hip fracture due to pain and difficulty weight-bearing, future studies could investigate the utility of tai chi in the later stages of hip fracture rehabilitation. While the intervention of motivational interviewing did show a promising result in one study [92], its effectiveness in addressing FoF in hip fracture patients’ needs further investigation.

Furthermore, in order to improve generalisability, future studies investigating FoF in hip fracture patients should consider including patients with some cognitive impairment as well as other co-morbidities. The existing literature has commonly excluded these patients, probably due to the difficulty of conducting research in these populations, including issues of consent. However, given that cognitive impairment and co-morbidities are extremely common in hip fracture patients [1] it is imperative for researchers to make an effort to include these groups to make their research more clinically useful.

Lastly, given the prevalence of FoF after hip fracture and consistent association with measures of physical function, we recommend that it should be included in the data collection in national hip fracture registries.

### Strengths and limitations of this review

This systematic review was undertaken in alignment with PRISMA guidelines which helped minimize bias and optimize the methodological quality of this study. The study protocol was pre-registered on PROSPERO to ensure that the researchers aligned with the set protocol throughout the course of the study, to minimize reporting bias. We only made a minor deviation from our protocol; we added the exclusion criteria for pilot or feasibility studies for research question four.

Two reviewers independently performed the database search, study screening, and selection to ensure robust data gathering and minimise error. Two independent reviewers undertook data extraction separately to ensure greater accuracy. Thorough and critical quality appraisal was completed using contemporary and stringent appraisal tools that have been developed by experts. We chose not to exclude studies with high risk of bias in order to provide a comprehensive overview of the existing literature and because the majority of intervention studies had a high risk of bias, however, this is a limitation of the current research. The reviewers were not blind to the names of the authors of included studies; however, there is no known bias from this as there are no affiliations or conflicts of interest. Lastly, as undertaking a meta-analysis was considered inappropriate; we did not formally measure and cannot account for any potential publication bias, which could be an important issue.

## Conclusion

This systematic review set out to synthesize existing literature on FoF after hip fracture in relation to four research questions: ‘what is the prevalence of FoF in hip fracture patients?’, ‘what are the psychometric properties of instruments used to measure FoF in hip fracture patients?’, ‘what measures of physical function or performance is FoF associated with in hip fracture patients?’ and ‘which interventions are effective in reducing FoF after hip fracture?’. This is the first systematic review to report FoF prevalence after hip fracture, which was consistently high, and to identify the trend that FoF appears to decrease as time passes post-fracture. Current evidence demonstrates that the FES-I and FFQ-R (6 and 15 item versions) are reliable and valid measures of FoF with a greater focus on falls efficacy and fear, respectively. Other commonly used instruments such as the short FES-I and ABC still need to be assessed in this population. This review found that FoF is consistently associated with measures of physical function or performance in hip fracture patients. However, the current literature does not definitively support any intervention to combat FoF in a hip fracture population, with important methodological limitations in many of the studies reviewed. To effectively guide clinical practice, there is a need for larger, higher quality randomised controlled trials that investigate targeted interventions with a sound theoretical base (for example, graded exposure), in both acute rehabilitation and community settings.

## Electronic supplementary material

Below is the link to the electronic supplementary material.

Supplementary File 1. Search Strategy.

Supplementary File 2. Quality Appraisal of Included Studies.

## Data Availability

The datasets used during the current study are available from the corresponding author on reasonable request.

## Abbreviations

CBT: cognitive behavioural therapy
FES-I: falls efficacy scale – international
FFQ-R: fear of falling questionnaire revised
FoF: fear of falling
OR: odds ratio
RCT: randomised controlled trial
SIQ: single item questionnaire.
