# Supplementary material for: A systematic review of fear of falling and related constructs after hip fracture: prevalence, measurement, associations with physical function, and interventions
Source: BMC Geriatr. 2023 Jun 23;23:385. doi: 10.1186/s12877-023-03855-9 (PMC10288814; doi:10.1186/s12877-023-03855-9)
Supplement: Supplementary file 1 — Search Strategy. [file 12877_2023_3855_MOESM1_ESM.docx]

**Supplementary Information (Online Only)**

**Supplementary File 1**

Search Strategy

The appropriate Boolean operators for each search engine were utilised as shown below.

EBSCO Health Databases

#1 (hip OR “neck of femur” OR “proximal femur” OR nof OR inter-trochanter* OR intertrochanter* OR “inter trochanter*” OR sub-trochanter* OR subtrochanter* OR “sub trochanter*”) n3 (fracture*)

#2 (fear* OR self-efficacy OR “self efficacy” OR confidence OR falls-efficacy OR “falls efficacy”) n5 (fall OR falls OR falling OR balance)

#3 Combine #1 AND #2

Scopus

#1 (hip OR “neck of femur” OR “proximal femur” OR nof OR inter-trochanter* OR intertrochanter* OR “inter trochanter*” OR sub-trochanter* OR subtrochanter* OR “sub trochanter*”) W3 (fracture*)

#2 (fear* OR self-efficacy OR “self efficacy” OR confidence OR falls-efficacy OR “falls efficacy”) W5 (fall OR falls OR falling OR balance)

#3 Combine #1 AND #2

PsychINFO

#1 (hip OR “neck of femur” OR “proximal femur” OR nof OR inter-trochanter* OR intertrochanter* OR “inter trochanter*” OR sub-trochanter* OR subtrochanter* OR “sub trochanter*”) adj3 (fracture*)

#2 (fear* OR self-efficacy OR “self efficacy” OR confidence OR falls-efficacy OR “falls efficacy”) adj5 (fall OR falls OR falling OR balance)

#3 Combine #1 AND #2

**Supplementary File 2**

*Quality Appraisal of Included Studies*

**Supplementary Table 1**

Quality appraisal of prevalence studies using Risk of Bias in Prevalence Studies tool

| **Item** | **1** | **2** | **3** | **4** | **5** | **6** | **7** | **8** | **9** | **10** | **Overall** |
| --- | --- | --- | --- | --- | --- | --- | --- | --- | --- | --- | --- |
| **Study** | **Target population** | **Sampling frame** | **Random selection** | **Non-response bias minimal** | **Direct data collection** | **Acceptable case definition** | **Reliable/ valid instruments** | **Same mode of data collection** | **Length of prevalence period** | **Appropriate numerator/ denominator** | **Risk of bias** |
| **Bower (2016)** | 1 | 1 | 1 | 1 | 0 | 0 | 0 | 0 | 0 | 0 | **Moderate Risk** |
| **Jaatinen (2022)** | 1 | 1 | 1 | 1 | 0 | 1 | 1 | 0 | 0 | 0 | **Moderate Risk** |
| **Koeda (2011)** | 1 | 1 | 1 | 0 | 0 | 1 | 1 | 0 | 0 | 0 | **Moderate Risk** |
| **Kornfield (2017)** | 1 | 1 | 1 | 0 | 0 | 1 | 1 | 0 | 0 | 0 | **Moderate Risk** |
| **Ungar (1986)** | 1 | 1 | 1 | 0 | 0 | 1 | 1 | 1 | 1 | 1 | **High Risk** |
| **Visschedijk (2013)** | 1 | 1 | 1 | 0 | 0 | 1 | 1 | 0 | 0 | 0 | **Moderate Risk** |
| 0 = Yes; 1 = No  Scoring: 0-3 Low Risk, 4-6 Moderate Risk, 7-9 High Risk (29) | | | | | | | | | | | |

**Supplementary Table 2**Quality appraisal of psychometrics studies using COSMIN tool

|  | **Bower (2015)** |  | **Visschedijk (2015)** |
| --- | --- | --- | --- |
| **Item** | **FFQ-R 15-item** | **FFQ-R 6 –item** | **FES-I** |
| **PROM Design** (1 – 35) | n/a | n/a | n/a |
| **Content Validity** (1 – 31) | n/a | n/a | n/a |
| **Structural Validity** |  |  |  |
| 1 Factor analysis | adequate | adequate | very good |
| 2 Rasch model | n/a | n/a | n/a |
| 3 Adequate sample size | very good | very good | adequate |
| 4 Other design/ statistical flaws | very good | very good | doubtful |
| Overall | Adequate | Adequate | Doubtful |
| **Internal Consistency** |  |  |  |
| 1 Statistic calculated for each scale | very good | very good | very good |
| 2 Continuous scores: Cronbach’s alpha | very good | very good | very good |
| 3 Dichotomous scores | n/a | n/a | n/a |
| 4 ‘Item Response Theory’ based scores | n/a | n/a | n/a |
| 5 Other design/ statistical flaws? | very good | very good | very good |
| Overall | Very Good | Very Good | Very Good |
| **Cross-cultural Validity** (1-4) | n/a | n/a | n/a |
| **Reliability** |  |  |  |
| 1 Patients stable in interim period | adequate | adequate | doubtful |
| 2 Appropriate time interval | inadequate | inadequate | inadequate |
| 3 Similar testing conditions | doubtful | doubtful | very good |
| 4 Continuous scores: ICC calculated | adequate | adequate | very good |
| 5 Dichotomous scores | n/a | n/a | n/a |
| 6 Ordinal scores: weighted kappa | n/a | n/a | n/a |
| 7 Ordinal scores: weighting scheme | n/a | n/a | n/a |
| 8 Other design/ statistical flaws | very good | very good | very good |
| Overall | Inadequate | Inadequate | Inadequate |
| **Measurement Error** | n/a | n/a |  |
| 1 Patients stable in interim period |  |  | doubtful |
| 2 Appropriate time interval |  |  | inadequate |
| 3 Similar testing conditions |  |  | very good |
| 4 Continuous scores: SEM/ SDC |  |  | very good |
| 5 Dichotomous scores |  |  | n/a |
| 6 Other design/ statistical flaws |  |  | very good |
| Overall |  |  | Inadequate |
| **Criterion Validity** (1-3) | n/a | n/a | n/a |
| **Hypotheses testing for Construct Validity** |  |  |  |
| 1 Clear comparator instrument | very good | very good | very good |
| 2 Measurement properties | very good | very good | very good |
| 3 Appropriate statistical method | very good | very good | very good |
| 4 Other design/ statistical flaws | very good | very good | very good |
| 5 Adequate description of subgroups | very good | very good | n/a |
| 6 Appropriate statistical method subgroups | very good | very good | n/a |
| 7 Other design/ statistical flaws (subgroups) | very good | very good | n/a |
| Overall | Very Good | Very Good | Very Good |
| **Responsiveness** (1 -13) | n/a | n/a | n/a |
| FFQ-R, fear of falling questionnaire revised; FES-I, falls efficacy scale international; PROM, patient-reported outcome measure; n/a, not applicable; ICC, intraclass correlation coefficient; SEM, standard error of measurement; SDC, smallest detectable change | | | |

**Supplementary Table 3**

*Quality appraisal of association studies using modified AXIS tool*

| **Item** | **1** | **2** | **3** | **4** | **5** | **6** | **7** | **8** | **9** | **10** | **11** | **12** | **13** | **14** | **15** | **16** | **17** | **18** | **19** | **20** | **21** | **22** | **23** |
| --- | --- | --- | --- | --- | --- | --- | --- | --- | --- | --- | --- | --- | --- | --- | --- | --- | --- | --- | --- | --- | --- | --- | --- |
|  | Clear aims | Study design | Sample size | Clear population | Sampling Frame | Sample represented | Address Non-response | Appropriate Measures | Reliable/ valid measures | Statistical significance | Statistics described | Basic data | Response rate | Describe non-responders | Internally consistent | All results | Justified conclusion | Limitations given | Funding/ conflicts | Ethics/ consent | Blinded assessors | Loss to follow-up | Adjusted confounders |
| **Abel (2020)** | Y | Y | N | Y | Y | N | DK | Y | Y | Y | Y | Y | DK | N | Y | Y | Y | N | N | Y | DK | Y | Y |
| **Briggs (2018)** | Y | Y | N | Y | DK | N | DK | Y | Y | Y | Y | Y | DK | N | Y | Y | Y | Y | N | Y | DK | NA | N |
| **Edgren (2013)** | Y | Y | N | Y | Y | Y | DK | Y | Y | Y | Y | Y | Y | N | Y | Y | N | Y | N | Y | DK | NA | N |
| **Ingermarsson (2000)** | Y | Y | N | N | Y | Y | DK | Y | Y | Y | N | Y | DK | N | Y | Y | N | N | N | DK | DK | Y | N |
| **Jaatinen (2022)** | Y | Y | N | Y | Y | N | DK | Y | Y | Y | Y | N | Y | N | N | Y | Y | Y | N | Y | DK | NA | Y |
| **Jellesmark (2012)** | Y | Y | N | Y | Y | N | DK | Y | Y | Y | Y | N | N | Y | Y | Y | Y | Y | N | Y | DK | NA | N |
| **Kline Mangione (2007)** | Y | Y | N | Y | DK | N | DK | Y | Y | Y | Y | Y | N | N | Y | Y | Y | Y | N | Y | DK | NA | N |
| **Kneiss (2015)** | Y | Y | N | Y | Y | N | DK | Y | Y | Y | Y | Y | DK | N | Y | Y | Y | Y | DK | Y | DK | NA | N |
| **Kronborg (2016)** | Y | Y | N | Y | Y | N | DK | Y | Y | Y | Y | Y | Y | N | Y | Y | Y | Y | N | Y | N | Y | N |
| **McKee (2002)** | Y | Y | Y | Y | Y | Y | DK | Y | N | Y | Y | Y | N | Y | Y | Y | Y | Y | N | Y | DK | N | Y |
| **Oude Voshaar (2006)** | Y | N | N | Y | Y | Y | Y | Y | Y | N | Y | N | Y | N | Y | Y | N | Y | N | Y | DK | N | Y |
| **Portegis (2012)** | Y | Y | N | Y | Y | Y | DK | Y | Y | Y | Y | Y | Y | N | N | Y | Y | Y | N | Y | DK | NA | Y |
| **Sihvonen (2009)** | Y | Y | N | Y | Y | N | DK | Y | Y | Y | N | Y | Y | N | Y | Y | N | Y | N | Y | DK | NA | N |
| **Whitehead (2003)** | Y | Y | N | Y | Y | N | Y | Y | Y | Y | N | N | N | N | Y | Y | N | Y | N | Y | DK | NA | N |
| **Willems (2017)** | Y | N | N | Y | Y | N | DK | Y | Y | Y | Y | Y | N | N | Y | Y | N | Y | DK | Y | DK | NA | Y |
| Y, yes; N, no; DK, don’t know; NA, not applicable  Note: Two negatively worded questions # 13 and # 19. | | | | | | | | | | | | | | | | | | | | | | | |

**Supplementary Table 4**

*Quality appraisal of intervention studies using RoB2.*

|  | | **Asplin (2017)** | **Beckmann (2021)** | **Birks (2003)** | **Crotty (2002)** | **Ko (2019)** | **Lee (2022)** | **Lockwood (2019)** | **O’Halloran (2016)** | **Peichl (2005)** | **Pfeiffer (2020)** | **Scheffers-Barnhoorn (2019)** | **Taraldsen (2019)** | **van Ooijen (2016)** | **Ziden (2008) Ziden (2010)** |
| --- | --- | --- | --- | --- | --- | --- | --- | --- | --- | --- | --- | --- | --- | --- | --- |
| 1.1  Randomised | | N | PN | Y | Y | N | Y | Y | Y | NI | Y | Y | Y | NI | NI |
| 1.2  Allocation concealed | | N | PY | Y | Y | N | PY | Y | Y | NI | PY | N | PY | NI | N |
| 1.3  Baseline differences | | N | Y | PN | N | N | PN | PN | PN | PN | PN | Y | PN | Y | N |
| ROB | | H.R. | S.C. | L.R. | L.R. | H.R. | L.R. | L.R. | L.R. | S.C. | L.R. | H.R. | L.R. | H.R. | H.R. |
| 2.1  Participant not blinded | | PY | Y | Y | Y | PY | PN | Y | Y | Y | Y | PN | Y | Y | PY |
| 2.2  Clinician not blinded | | PY | Y | PY | PY | PY | Y | Y | Y | Y | Y | Y | Y | Y | PY |
| 2.3  Deviation from intervention | | NI | NI | NI | NI | NI | NI | PN | N | NI | NI | NI | Y | PN | NI |
| 2.4 | | NA | NA | NA | NA | NA | NA | NA | NA | NA | NA | NA | PY | NA | NA |
| 2.5 | | NA | NA | NA | NA | NA | NA | NA | NA | NA | NA | NA | N | NA | NA |
| 2.6  Intention-to-treat | | NI | Y | NI | Y | N | PY | Y | N | PN | Y | Y | Y | N | N |
| 2.7 | | PY | NA | PY | NA | NI | NA | NA | NA | PN | NA | NI | PN | PN | Y |
| ROB | | H.R. | S.C. | H.R. | S.C. | H.R. | H.R. | L.R. | H.R. | S.C. | S.C. | S.C. | H.R. | S.C. | H.R. |
| 3.1  All data | | N | N | N | PY | PN | N | N | N | PN | N | N | N | N | N |
| 3.2  Bias by missing data | | N | Y | PN | NA | PN | PN | PY | PN | N | Y | PN | PN | PN | PN |
| 3.3 | | PY | NA | PY | NA | PY | PY | NA | PY | PY | NA | PY | PY | PY | PN |
| 3.4 | | PY | NA | PN | NA | PY | PN | NA | PN | PY | NA | PN | PY | PN | NA |
| ROB | | H.R. | L.R. | S.C. | L.R. | H.R. | S.C. | L.R. | S.C. | H.R. | L.R. | S.C. | H.R. | S.C. | L.R. |
| 4.1  Inappropriate measures | | N | N | PN | N | N | N | N | N | N | N | N | N | N | PN |
| 4.2  Between groups differences | | N | PN | N | PN | PN | PN | N | PN | PN | PN | PN | PN | PN | PN |
| 4.3  Assessor not blinded | | Y | N | PY | Y | Y | PY | Y | Y | PY | Y | PN | Y | Y | Y |
| 4.4 | | PY | NA | PY | Y | PY | PY | PY | PY | PY | PY | NA | PY | PY | PY |
| 4.5 | | PN | NA | PN | PN | PN | PN | PN | PN | PN | PN | NA | PN | PN | PN |
| ROB | | S.C. | L.R. | S.C. | S.C. | S.C. | S.C. | S.C. | S.C. | S.C. | S.C. | L.R. | S.C. | S.C. | S.C. |
| 5.1  Data analysis pre-specified | | NI | NI | NI | NI | NI | NI | PY | PY | NI | NI | NI | N | NI | NI |
| 5.2 | | NI | NI | NI | NI | NI | N | PN | PN | NI | NI | NI | PN | NI | NI |
| 5.3 | | NI | NI | NI | NI | NI | NI | N | N | NI | NI | NI | PN | NI | NI |
| ROB | | S.C. | S.C. | S.C. | S.C. | S.C. | S.C. | L.R. | L.R. | S.C. | S.C. | S.C. | S.C. | S.C. | S.C. |
| Overall R.O.B Judgement | | High Risk | Some Concerns | High Risk | Some Concerns | High Risk | High Risk | Some Concerns | High Risk | High Risk | Some Concerns | High Risk | High Risk | High Risk | High Risk |
|  | Y, yes; PY, probably yes; N, no; PN, probably no; NA, not applicable; NI, no information; ROB, risk of bias; H.R., high risk; S.C., some concerns; L.R., low risk | | | | | | | | | | | | | | |
